# KRAS and BRAF mutational status in colon cancer from Albanian patients

**DOI:** 10.1186/s13000-014-0187-7

**Published:** 2014-09-30

**Authors:** Daniela Martinetti, Rosario Costanzo, Shahin Kadare, Mehdiu Alimehmeti, Cristina Colarossi, Vincenzo Canzonieri, Massimiliano Berretta, Lorenzo Memeo

**Affiliations:** IOM Ricerca Srl, Catania, Italy; Morphopathology Laboratories, Tirana, Albania; Department of Pathology, University of Tirana, Tirana, Albania; Department of Experimental Oncology, Mediterranean Institute of Oncology, Via Penninazzo 7, 95029 Viagrande, Catania, Italy; Division of Pathology, Centro di Riferimento Oncologico, Aviano, Italy; Department of Clinical Oncology, Centro di Riferimento Oncologico, Aviano, Italy

**Keywords:** Colorectal cancer, KRAS, BRAF, Albania

## Abstract

**Background:**

Numerous clinical studies have shown that anti-EGFR therapies are effective only in a subset of patients with colorectal cancer. Mutations in the KRAS and BRAF genes have been confirmed as negative predictors of the response to EGFR-targeted therapies.

In this study we evaluated KRAS and BRAF status in 159 colorectal cancer samples obtained from the University of Tirana.

**Methods:**

We evaluated KRAS mutations in codons 12, 13, 61, 146 and in codon 600 of BRAF by direct sequencing. 90 patients were male (57%) and 69 female (43%); the patients’ ages ranged from 17 to 85 (median 61.7). 24 patient were stage I, 36 stage II, 84 stage III and 15 stage IV.

**Results:**

Out of the 159 cases, 28 (17,6%) showed KRAS mutation (13 G12D, 4 G12C, 4 G12V, 3 G12A, 2 G13 D, 1 G12S and 1 A146T), and 10 (6,3%) showed BRAF mutation (all V600E). No significant correlations between KRAS and BRAF mutations and various clinicopathological parameters was found.

This is the first report of KRAS and BRAF status in Albanian patients with colorectal carcinoma (CRC) and though the relatively small sample size might not provide enough statistics power.

**Conclusions:**

The results of KRAS and BRAF mutation analysis could be used in the selection of patients for anti-EGFR therapy.

**Virtual Slides:**

The virtual slide(s) for this article can be found here: http://www.diagnosticpathology.diagnomx.eu/vs/13000_2014_187

## Background

Colorectal cancer (CRC) is the third most common malignancy in men and women with an incidence of 1,234,000 cases contributing 10% of the total burden [1]. The incidence rates of colon cancer vary in various geographic locations with highest rates estimated in Australia/New Zealand and Western Europe [2].

The development of CRC is a multistep process that occurs because of the accumulation of several genetic alterations, including chromosomal abnormalities, gene mutations, and epigenetic modifications involving several genes that regulate proliferation, differentiation, apoptosis, and angiogenesis [3,4].

Of the various genetic alterations, an important molecular target for metastatic CRC treatment is the epidermal growth factor receptor (EGFR). EGFR, a tyrosine kinase receptor belonging to the ErbB family, has been found to play a significant role in the pathogenesis of CRC by inducing downstream signaling pathways, such as the phosphatidylinositol-3-kinase/Akt and RAS/RAF/MAP-activated protein kinase (MAPK) pathways.

The EGFR is overespressed in about 50-80% of CRCs and has been the focus of a new drug development [5] and antibodies targeting EGFR, such as cetuximab and panitumumab, have been examined for therapeutic efficacy in CRC patients [6].

Oncogenic activation of signaling pathways downstream of the EGFR, as induced by mutated KRAS or BRAF, is important for the progression of colorectal cancer [7].

In human CRC, mutations in the *KRAS* gene have a frequency of around 30%-40% and were linked to poor outcomes, whereas mutations of the *BRAF* gene, a downstream molecule of KRAS, occur in only 5%-10% of patients with sporadic diseases. Mutations in the *KRAS* and *BRAF* genes are frequently found to be mutually exclusive in colorectal cancer [8,9].

Recently, any activating mutations in the *KRAS* gene has been proved to be predictor of response to epidermal growth factor receptor-targeted therapies, such as cetuximab and panitumumab, for patients with metastatic colorectal cancer [10].

Different somatic missense mutations in codons 12, 13, 61 and 146 are relevant for therapeutic efficacy of anti-EGFR therapy [10-14]. In addition, among colorectal tumors carrying wild-type *KRAS*, mutation of *BRAF* or *NRAS* or *PIK3CA* or loss of *PTEN* expression may be associated with resistance to EGFR-targeted monoclonal antibody treatment, although these additional biomarkers require further validation before incorporation into clinical practice [15].

Activating mutations of these oncogenes are point mutations (missense mutations) and they arise early during the development of colorectal cancer [16]. The activating mutations in *KRAS* oncogene are found mostly in codons 12 and 13 (>90%) but also affect codons 61 and 146 [12,13]. The most frequently observed types of mutations are G > A transitions, G > T and G > C transversions [17]. Identification of somatic-activating mutations of *BRAF* has been reported in various cancers, with by far the most common mutation being a 1799 T > A transversion leading to a Val600Glu (V600E) substitution [9].

Recent studies have demonstrated that a wild type BRAF is also required for response to panitumumab or cetuximab, suggesting that BRAF evaluation should be used together with KRAS for selecting the patients who could benefit from the anti-EGFR therapy [18,19].

In the present study, we detected mutations of KRAS and BRAF proto-oncogenes in tumoral tissue specimens in CRC patients of the Albanian population.

Correlations with various clinicopathological characteristics of patients were further analyzed. To our knowledge, we are the first to report the frequency and type of KRAS and BRAF mutations in Albanian patients with advanced CRC in order to introduce targeted therapy in the therapeutic modalities for management of this cancer in Albania.

Further researches are needed to determine how the racial differences and etiological factors can influence the spectrum and frequency of KRAS and BRAF mutations between different populations.

## Methods

### Patients and specimens

Tumor specimens used in this study were obtained from 159 CRC consecutive patients who underwent tumor resection at Tirana University Hospital during the period 2012–2013.

The study included 159 patients with histopathologically proven colorectal cancer; two pathologists independently confirmed the diagnosis.

Tumor stage was classified according to the Tumor, Node and Metastases (TNM) classification of the Union for International Cancer Control (UICC) staging.

The present study received the IRB approval from University of Tirana (#12; 21/02/2014).

### Histological examination

Tumors were classified as well-, moderately or poorly differentiated adenocarcinoma in accordance with the World Health Organization Classification (Jass JR, Sobin LH. Histological typing of intestinal tumors. In World Health Organization, ed. International Histological Classification of Tumors, 2nd edn. Berlin: Springer, 1989; 29–40). Mucinous tumors were separately classified.

Additional histological features such as necrosis, tumor vascular invasion, stromal desmoplastic reaction and infiltrative versus expansive pattern of growth, have been evaluated but not considered as variable parameters for statistical analysis.

### DNA isolation

Sections (5 micron) were cut from paraffin-embedded tumor tissue blocks and stained with haematoxylin & eosin (H&E) for histopathological examination.

For DNA isolation, 5 sections, each of 4 micron thickness, were used for each case. The H&E section was used as a reference and tumor tissue was macrodissected from the normal colonic epithelium and scraped off.

Genomic DNA was extracted from formalin-fixed paraffin-embedded (FFPE) tissue samples using the QIAamp DNA FFPE tissue kit (Qiagen) according to the manufacturer’s recommendations and was amplied by PCR at KRAS exon 2, 3 and 4 and BRAF exon 15.

### Analysis of KRAS and BRAF mutations

Mutation analysis of KRAS codons 12, 13, 61, 146 and BRAF codon 600 was carried out by direct sequencing of amplified PCR products.

PCR was performed using 50 ng genomic DNA as template. Each mixture contained 8 pmol of each primer. Primers were purchased from Roche Dagnostics Spa, Monza, Italia.

The reactions were performed in 1X GeneAmp 10X PCR Buffer II (Applied Biosystems), 0.25 μmol/L dNTPs, 2 mmol/L MgCl2 solution, and 1.25 U AmpliTaq DNA Polymerase (Applied Biosystems, CA USA).

The amplification reactions were as follows: an initial denaturation cycle of 95°C for 5 min; 45 cycles of denaturation (95°C for 30 s), annealing (60°C for 30 s for KRAS exon 2, 55°C for 30 s for KRAS exon 3, 4 and 57°C for 30 s for BRAF exon 15), and elongation (72°C for 1 min); and a final extension cycle at 72°C for 5 min.

The PCR products were purified with 1 ml ExoI/SAP (37°C for15 minutes, then 85°C for 15 minutes) and were then sequenced directly on both strands using the BigDyeH Terminator v1.1 cycle sequencing kit (Applied Biosystems) according to manufacturer’s protocol and analysed by the ABI 3500 Genetic Analyzer (Applied Biosystem, CA, USA).

## Results

Of the 159 patients, 90 were male and 69 female; ranged from 17 to 85 years of age (median 61.7). 24 patients were stage I, 36 stage II, 84 stage III and 15 stage IV.

KRAS mutational status was tested in 159 clinical samples of which 28 (17,6%) harboured at least one mutation at codon 12, 13 or 146.

Specific nucleotide and codon changes detected are listed in Table 1.

**Table 1:** **Frequency of Mutations in KRAS codon 12, 13, 61 and 146 (N = 159)**

**Nucleotide change**	**Aminoacid change**	**N. of mutated cases (%)**
KRAS codon 12		
c.35G > A	p.G12D	13 (8.1)
c.34G > A	p.G12S	1 (0.6)
c.35G > T	p.G12V	4 (2.5)
c.34G > T	p.G12C	4 (2.5)
c.35G > C	p.G12A	3 (1.9)
KRAS codon 13		
c.38G > A	p.G13D	2 (1.3)
KRAS codon 61		
/	/	0
KRAS codon 146		
c.436G > A	p.A146T	1 (0.6)

**Figure 1:**
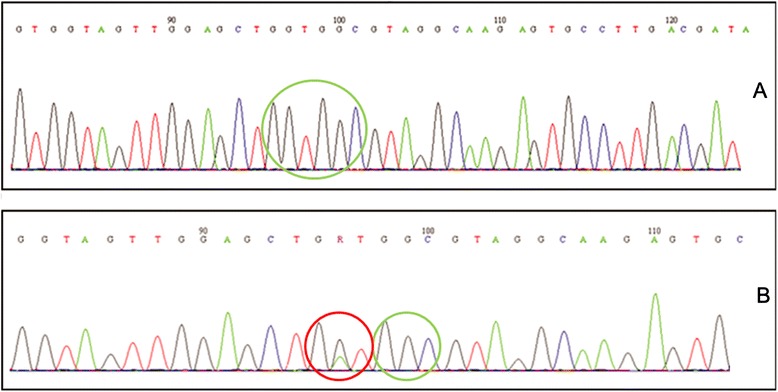
**Sequencing electropherogram of KRAS exon 2. (A) Wild-type. (B) G12D mutation.**

Mutations at codon 12 are the more frequent ones, followed by codon 13 and 146 ones. No point mutation was detected in KRAS at codon 61. 25 samples showed a mutation at codon 12, 2 at codon 13 and 1 at codon 146. The incidence of KRAS mutation was similar in men and women. The predominant mutations were G > A transition while the most frequent mutation was G12D (8.1% of all mutations). Representative electropherograms of KRAS wild type and G12D are shown in Figure 1.

Of the 131 KRAS wild type samples, 10 (6,3%) harboured a mutation at codon 600 in exon 15 of BRAF (V600E) (Table 2).

**Table 2:** **Frequency of BRAF Mutations in Tumor Wild type for KRAS codon 12, 13, 61 and 146 (N = 131)**

**Nucleotide change**	**Aminoacid change**	**No. of mutated cases (%)**
BRAF codon 600		
c.1799 > A	p. V600E	10 (6.3%)

**Figure 2:**
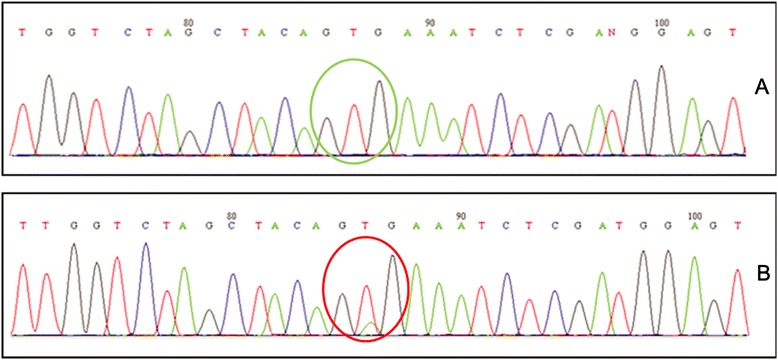
**Sequencing electropherogram of BRAF exon 15. (A) Wild-type. (B) V600E mutation.**

Representative electropherograms of BRAF wild type and with a V600E mutation are shown in Figure 2. In addition, our data confirmed that mutations in KRAS and BRAF are mutually exclusive.

All mutations found had previously been described to be oncogenically active and were found in the COSMIC (catalog of somatic mutations in cancer) database (Sanger Institute, Cambridge, UK).

No association between KRAS and BRAF mutations and various clinicopathological features such as age, gender, tumor differentiation, UICC classification was found (Table 3).

**Table 3:** **Correlation between KRAS, BRAF mutations and clinicopathological parameters in colorectal cancer (%)**

**Terms**	**All**	**Wild type**	**KRAS Mutation**	**BRAF Mutation**
No.of patients	159	122	28 (17,6%)	10 (6,3%)
Median age	61,7			
Gender				
Male	90	69	18 (20%)	3 (3,3%)
Female	69	53	10 (14,5%)	7 (10,1%)
Differentiation				
Poor	12	11	/	1 (8,3%)
Moderate	76	57	17 (22%)	3 (3,9%)
Mucinose	16	12	3 (18,7%)	1 (6,2%)
Well	52	39	8 (15,3%)	5 (9,6%)
UICC classification				
I	24	17	5 (20,8)	2 (8,3%)
IIA	36	26	7 (19,4)	3 (8,3%)
IIIA	10	9	1 (10%)	/
IIIB	70	55	11 (16%)	5 (7.1%)
IIIC	4	3	1 (25%)	/
IV	15	12	3 (20%)	/

## Discussion

The epidermal growth factor receptor (EGFR) plays a key role in the development and progression of CRC. It triggers a downstream signaling cascade such as RAS-RAF-MAPK and PI3K-AKT pathway, which are involved in cell proliferation, differentiation, survival and invasion. Among the activating mutations downstream of EGFR, KRAS and BRAF oncogenes participate in the MAPK pathway that mediates cellular response to growth signals [20]. This cascade is activated by the EGFR [21] that is overespressed in 50-80% of colorectal tumors and therefore represent a suitable target for the anticancer therapies with monoclonal antibodies as cetuximab and paninutumab [22,23]. These molecules bind to the extracellular domain of EGFR leading to the inhibition of its downstream signaling.

The anti-EGFR therapies have shown to be effective only in a subset of patients with colorectal cancer [24]. To optimize the benefits and to reduce the risks of anti-EGFR therapies, the EGFR, as well as the molecules involved in its pathway, has been evaluated as potential marker to predict the treatment outcomes. Recents studies have demonstrated that mutations in the KRAS gene negatively predict the response to EGFR-targeted therapies in patients with metastatic colorectal cancer [25,26]. The KRAS mutations that are responsible for the synthesis of a permanently active KRAS protein [27,28] are predominately identified in the 12 and 13 codon of the gene [28]. Despite having a wild type KRAS, only 40-60% of the patients will respond to treatment [29]. The identification of the other important molecular determinants of response is therefore of an outmost importance.

The BRAF-activating mutations have been reported in various type of cancer: melanoma (70% of cases), thyroid (30-70%), ovarian (15-30%) and colorectal cancer (5-10%) [30,31]. All BRAF mutations occur within the kinase domain resulting in an elevated kinase activity of the BRAF protein. The p. Val600Glu (V600E) mutation is the most common mutation in the BRAF gene, found in approximately 80% of the cases [9,19]. Certain studies have demonstrated that wild type BRAF is required for response to panitumumab or cetuximab, suggesting that BRAF should also be tested together with KRAS to select the patients who are most likely to benefit from the anti-EGFR therapy [19,32,33]. Our objective was the determine the frequency of most common mutations in KRAS gene (p.G12D, p.G12V, p.G12A, p.G12C, p.G12S, p.G12R, p.G13D, p.Q61H, p.Q61L, p.Q61R, p.A146T, p.A146V, p.A146P) together with the BRAF V600E mutation in Albanian patients with metastatic colorectal cancer.

In our study, we have evaluated KRAS and BRAF mutational status in 159 Albanian CRC patients using direct sequencing. The present study is first to provide data on frequency and type of KRAS and BRAF mutations of colorectal cancer in Albanian population; no data is present in the literature about this incidence.

In addition, we also tried correlate the presence of KRAS and BRAF mutations at codon 12, 13, 61, 146 and 600 with various clinicopathological features such as age, gender and grade as shown in Table 3.

In the present study, we did not find any significant correlations between these molecular events and various clinicopathological features, which may be partly attributable to the relatively small sample size.

In summary, our study reports that the incidence of KRAS mutation in Albanian colorectal cancer patients is less frequent when compared with the data from literature (35-50%) [14,34-37].

This is probably due to the different methodology since we used direct sequencing and to small sample size.

Studies from various countries have analyzed the frequency of the type of KRAS point mutation in CRC. Most of the authors have identified the G > A transition as the most frequently found type of KRAS mutation [38,39]. In the current study, the G > A transition appeared also to be the predominant mutation, followed by G > T transversion. Among mutations in codons 12, the substitution of glycine with aspartate has been reported as the most frequent change. In accordance with our data, previous studies have usually identified the glycine to aspartate transition on codon 12 (p.G12D) as the most frequent mutation of KRAS [40-48].

The percentage of BRAF mutation is similar to the published data reporting the BRAF V600E mutation in the range of 5 to 10% [19,49].

Although the mutations in KRAS are considered to be a highly specific negative marker of response to cetuximab and panitumumab, the selection of patients for anti-EGFR treatment on such basis is not sensitive enough. Moreover, it has been reported that patients with activating mutations in RAS, in addition to KRAS exon 2, do not benefit from combined panitumumab plus FOLFOX4 chemiotherapy [10].

Therefore, the BRAF mutational status is of utmost importance to be verified as another molecular determinant of response to anti-EGFR targeted monoclonal antibody therapy.

In our group, we found 10 patients with the V600E mutation in BRAF. These 10 patients represent 6.3% of all tested patients.

All 10 patients with the V600E mutation in BRAF were wild-type KRAS, and no BRAF mutations were found in patients with a mutated KRAS genotype. This also is in concordance with previously published observations by other authors that mutations in KRAS and BRAF are mutually exclusive [8].

## Conclusions

In conclusion, we observed a frequency of 17,6% for mutations in exon 2, 3 and 4 of the KRAS oncogene, predominantly in codon 12. The G > A transition and G > T transversion were the most frequently observed mutations, with the G > T transversion confined to codon 12.

Summing up the results about the KRAS and the BRAF mutation carriers from our study, the portion of potentially non-responsive patients for the anti-EGFR treatment is 23,3%.

Thus, the anti-EGFR therapy could be beneficial for the majority of Albanian population.

The results of this study indicate that the types of KRAS mutations from CRC in Albania are similar to other countries but with a lower frequency.

A limitation of this study is the absence of data on NRAS mutations, considering that patients with RAS mutations (KRAS or NRAS) do not respond to anti-EGFR therapy.

These data should be confirmed on a larger study group and in prospective studies in order to determine whether these mutations contribute to progression of CRC.

### Consent

Written informed consent was obtained from the patients for the publication of this study and any accompanying images.

## Abbreviations

CRC: Colorectal cancer
EGFR: Epidermal growth factor receptor
TNM: Tumor, node and metastases
UICC: Classification of the Union for International Cancer Control staging
H&E: Haematoxylin & eosin
FFPE: Formalin-fixed paraffin-embedded
